# Socioeconomic indicators of health inequalities and female mortality: a nested cohort study within the United Kingdom Collaborative Trial of Ovarian Cancer Screening (UKCTOCS)

**DOI:** 10.1186/s12889-015-1609-5

**Published:** 2015-03-17

**Authors:** Katharine Bailey, Andy Ryan, Sophia Apostolidou, Evangelia Fourkala, Matthew Burnell, Aleksandra Gentry-Maharaj, Jatinderpal Kalsi, Max Parmar, Ian Jacobs, Hynek Pikhart, Usha Menon

**Affiliations:** Department of Women’s Cancer, Institute for Women’s Health, University College London, Maple House, 149 Tottenham Court Road, London, W1T 7DN United Kingdom; Medical Research Council Clinical Trials Unit at University College London, London, WC2B 6NH United Kingdom; Faculty of Medical and Human Sciences, University of Manchester, Manchester, M13 9PT United Kingdom; Department of Epidemiology and Public Health, University College London, London, W1T 7DN United Kingdom

**Keywords:** UKCTOCS, Mortality, Socioeconomic, Education, IMD

## Abstract

**Background:**

Evidence is mounting that area-level socioeconomic indicators are important tools for predicting health outcomes. However, few studies have examined these alongside individual-level education. This nested cohort study within the control arm of the United Kingdom Collaborative Trial of Ovarian Cancer Screening (UKCTOCS) assesses the association of mutually adjusted individual (education) and area-level (Index of Multiple Deprivation-IMD 2007) socioeconomic status indicators and all-cause female mortality.

**Methods:**

Participants resident in England who had completed both baseline (Wave 1) and follow up (Wave 2) questionnaires were included. Follow-up was through the Health and Social Care Information Centre with deaths censored on 31st December 2012. IMD, education and a range of covariates were explored. Cox regression models adjusted for all covariates were used. Sensitivity analysis using imputation was performed (1) including those with missing data and (2) on the entire cohort who had completed the baseline questionnaire.

**Results:**

Of the 54,539 women resident in England who completed both Wave 1 and Wave 2 questionnaires, 4,510 had missing data. The remaining 50,029 women were included in the primary analysis. Area-level IMD was positively associated with all-cause mortality for the most deprived group compared to the least deprived (HR=1.42, CI=1.14-1.78) after adjusting for all potential confounders. Sensitivity analyses showed similar results with stronger associations in the entire cohort (HR=1.90, CI=1.68-2.16). The less educated an individual, the higher the mortality risk (test for trend p=<0.001). However, the crude effect on mortality of having no formal education compared to college/university education disappeared when adjusted for IMD rank (HR=1.08, CI=0.93-1.26).

**Conclusion:**

Women living in more deprived areas continue to have higher mortality even in this less deprived cohort and after adjustment for a range of potential confounders.

**Trial Registration:**

This study is registered as an International Standard Randomised Controlled Trial, number ISRCTN22488978.

**Electronic supplementary material:**

The online version of this article (doi:10.1186/s12889-015-1609-5) contains supplementary material, which is available to authorized users.

## Background

It has been repeatedly shown that socioeconomic status can have a detrimental effect on health. [1-6] Socioeconomic differences in health outcomes including mortality are one of the most consistent findings in epidemiology [7]. Income [8,9], occupation [10,11], and education [12-14] are all among major determinants of population distribution of health. More recently, researchers have become increasingly interested in area-level socioeconomic indicators of health inequalities and in whether they play a role over and above individual-level socioeconomic indicators [15-22]. Data used for such analyses are often of insufficient size. Multi-level analysis focusing on the role of socioeconomic determinants of mortality (both all-cause and cause-specific) is rare [19]. This has resulted in growing consensus for the need of multi-level analysis in data from larger cohorts [15,23-25]. We report on the association between individual-level education and area-level measures of social position and all-cause and cause-specific mortality in women aged 50–74 years in the control arm of the United Kingdom Collaborative Trial of Ovarian Cancer Screening (UKCTOCS) adjusting for a range of individual-level covariates, health behaviours and comorbidity. The advantage of this analysis is that it uses recent data from 2001–12 and is of larger size than other UK and most European-based studies on the relative role of individual-level education and area-based measures of socioeconomic position. The aim of the study is to assess whether there are associations between area-level deprivation and mortality over and above individual-level socioeconomic measures.

## Methods

UKCTOCS is a randomised controlled trial set in 13 National Health Service Trusts in England, Wales and Northern Ireland. The aim is to assess the impact of screening on ovarian cancer mortality by comparing participants randomised to annual screening with serum CA125 or transvaginal ultrasound (study arm) to those with no intervention (control arm) [26]. Inclusion criteria were age (50–74) and postmenopausal status. Exclusion criteria included bilateral oophorectomy, increased risk of familial ovarian cancer, previous ovarian malignancy, active non-ovarian malignancy and participation in other ovarian cancer screening trials. The study was approved by the UK North West Multicentre Research Ethics Committees (North West MREC 00/8/34) along with site specific approval from the local regional ethics and the Caldicott guardians of the primary care trusts [27]. Written informed consent was obtained from all volunteers.

Between 2001 and 2005, 1,243,312 women were invited at random from those that were eligible from 27 local health authority registers [27]. These were located around 13 regional centres – Belfast, Bristol, Cardiff, Derby, Gateshead, Liverpool, Manchester, Middlesbrough, North Wales, Nottingham, Portsmouth, Royal Free and St Bartholomew’s Hospitals in London. Potential participants were sent invitations to participate. If they accepted, they were given an appointment at a local trial centre where they viewed an information video, participated in a group discussion and had a private, one to one session with a research nurse before signing the consent form. The overall acceptance rate was 24.8% [27] with 202,638 women successfully recruited [26]. Only women residing in England were included in this analysis.

Participants completed a baseline questionnaire when they entered the study (Wave 1) and were sent a follow−up questionnaire approximately 3.5 years later (Wave 2). The baseline questionnaire included questions on medical history, demographic and physical characteristics whilst the follow up questionnaire included questions on medical history, education, lifestyle (including alcohol consumption and smoking habits) and outlook on life. Index of Multiple Deprivation (IMD) 2007 [28] was calculated using the post code. The IMD is made up of 7 domains in which 38 indicators, appropriately weighted, are used to calculate a deprivation score. Each postcode has its own individual score. The volunteers’ deprivation scores, varying from 0.72 to 85.46, were divided into six groups ranging from the least deprived to the most deprived.

All volunteers were flagged for cancer registrations/deaths through the appropriate national agency, the Health and Social Care Information Centre (HSCIC). The updated cancer registration listings for this analysis were received on 3rd June 2013. Death registration and cause of death information was obtained from HSCIC every quarter and the dataset updated accordingly. Cause of death was recorded using the World Health Organisation’s International Classification of Diseases 10th revision (ICD10). In the UK, majority of the deaths are registered within three months, hence events were censored on the 31st December 2012, 3 months prior to the last death certificates update in March 2013 from HSCIC.

### Variables

STATA 12 statistical package was used for the analysis. Participants in this nested cohort study were women in the control group (to ensure that screening undertaken in the trial had no impact on mortality) who were resident in England (as the IMD 2007 [28] excludes Wales and Northern Ireland). The Northern Ireland Deprivation Measure 2010 [29] and the Welsh IMD 2008 [30] have not been used, as the differing criteria between the three measures would make comparisons untenable. As the education exposure variable was only included in the follow up questionnaire, participants who completed the baseline questionnaire but not the follow up questionnaire were excluded.

The main outcome was all-cause mortality during the study period. Additional outcomes were ‘cardiovascular disease’ (CVD) and ‘cancer’ specific mortality. Cause of death was recorded using the ICD10–‘Ischaemic heart disease and diseases of the circulatory system’ and ‘Neoplasms’ respectively. Variables explored were IMD, age, ethnicity, Hormone Replacement Therapy (HRT) use, Body Mass Index (BMI) collected at Wave 1 and education, alcohol consumption, smoking, change in participants skirt size between their early 20’s and on completion of the follow-up questionnaire, being or having been treated for any of the following conditions; high blood pressure, heart disease, high blood cholesterol, diabetes, rheumatoid arthritis, osteoarthritis, stroke, osteoporosis, or none of the above conditions collected at Wave 2. Participants with missing data on covariates, miscoded height (<120 cm and >210 cm) and weight (<30 kg and >200 kg) or incorrect dates (i.e. follow-up questionnaire completion date or death date that preceded the date the follow up questionnaire was printed) were excluded.

Baseline characteristics of the study participants were examined. 95% confidence intervals were used throughout. The date of study entry was the date that the completed follow-up questionnaire was received by the Gynaecological Cancer Research Centre (GCRC), University College London (UCL) (or date printed, in the event where the date received had not been recorded). The date of study exit was censored on the 31 December 2012 or the death date as provided by HSCIC. A follow up time was created for those who completed the follow up questionnaire, from the study entry date to study exit date.

### Statistical analysis

Mortality rates for all covariates were calculated and the statistical significance of the association between covariates and mortality was assessed. A Cox regression model was used to analyse the hypotheses, [1] those living in more deprived areas in England will have a higher mortality risk than those living in less deprived areas and [2] those with lower educational attainment will have a higher mortality risk than those with higher educational attainment.

The regression models were then adjusted for age and all other covariates from the baseline and follow-up questionnaires. Country of birth was not included as it did not have a statistically significant association with mortality. To account for potential geographical clustering (within-cluster correlation) of the data, standard errors were calculated using a cluster-robust estimator. Geographic clusters were defined using the Super Output Area 1 codes [31], linked to provided postcodes, resulting in 6954 clusters. A test for trend was performed on all models and interaction between the main exposures of education and IMD was assessed using the likelihood-ratio test. The Kaplan-Meier method was used to obtain survival curves to compare the death rates by education and IMD. The least deprived group of IMD rank and those with college or university education were used as the reference categories for all regression analyses.

Further analysis was done for cancer and CVD mortality. The relationship between education and IMD rank and CVD and cancer was analysed using Cox regression. The regression models were adjusted for age and a test for trend was performed.

Sensitivity analysis was performed on [1] the entire English cohort who had completed the baseline questionnaire using the smaller number of variables available (Wave 1) and [2] participants who had completed the follow-up questionnaire including those with missing and miscoded data (Wave 2). Multiple imputation of the missing data was performed because sensitivity analysis did not show any substantial differences in distribution of covariates among those with no missing data and those with some missing data. Ten imputations were considered sufficient and 10 imputed datasets were produced using the multiple imputation command in STATA version 12. Appropriate commands relevant for imputed data were then used in the regression analysis.

## Results

**Figure 1:**
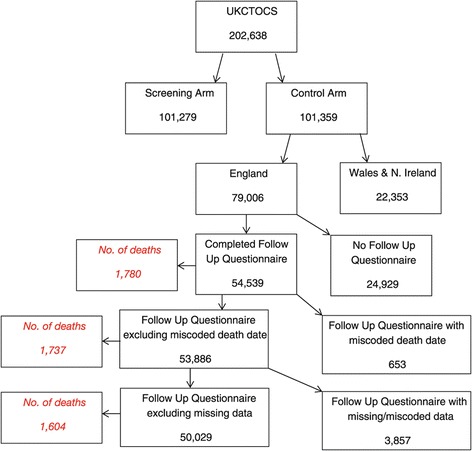
**Derivation of the analytical sample.**

There were 79,006 participants in the UKCTOCS control group who were resident in England. 54,539 completed both baseline and follow-up questionnaires. 4510 were excluded due to incorrect dates (653), missing data (3,395) and miscoded height and weight (462). The remaining 50,029 women were included in the final analysis (Figure 1).

The characteristics of the study participants are detailed in Table 1. All were resident in England but only 89.5% were born in England with the remaining 2.1% born in Scotland, 1.4% in Wales, 0.5% Northern Ireland, 1.3% in Irish Republic and 5.3% elsewhere. The median age was 60 (interquartile range 10) and 98% were Caucasian. The median BMI was 26. Twenty percent had been educated to college or university level. Sixty five percent of women were in the least two deprived categories of the six deprivation categories.

**Table 1:** **The association between all-cause mortality, education, IMD rank and all other variables in a mutually adjusted model, n=50,029**

	***Number***	***%***	***HR (95% CI)***	***P-value***	***P-value for trend***
***Education Level***					
College/University	10300	20.6	1.00		<0.001
Other formal qualification	24689	49.4	1.04 (0.90-1.20)	0.580	
No formal qualification	15040	30.1	1.08 (0.93-1.26)	0.334	
***IMD Rank***					
1=Least deprived (0–9)	16672	33.3	1.00		<0.001
2 (10-19)	16950	33.9	1.14 (1.00-1.30)	0.048	
3 (20-29)	7797	15.6	1.10 (0.94-1.29)	0.230	
4 (30-39)	4212	8.4	1.44 (1.21-1.72)	<0.001	
5 (40-49)	2317	4.6	1.42 (1.14-1.77)	0.002	
6=Most deprived (50+)	2081	4.2	1.42 (1.12-1.81)	0.004	
***Age***			1.10 (1.09-1.11)	<0.001	-
***Ethnicity***					
White	48904	97.8	1.00		-
Black	442	0.9	0.85 (0.48-1.51)	0.579	
South Asian	257	0.5	0.14 (0.20-1.00)	0.05	
Other	426	0.9	0.56 (0.25-1.25)	0.158	
***BMI Kg/M*** ^***2***^ ***(Continuous)***			1.00 (0.99-1.01)	0.872	-
***BMI Categories Kg/M*** ^***2***^					
Normal (20-24)	20,385	40.75	1.00		0.012
Underweight (11-19)	1,866	3.73	1.20 (0.94-1.53)	0.149	
Overweight (25-29)	18,376	36.73	0.95 (0.84-1.07)	0.374	
Obese (30-39)	8,660	17.31	1.00 (0.85-1.17)	0.983	
Morbidly Obese (40+)	742	1.48	1.46 (1.03-2.07)	0.033	
***HRT (Current at baseline)***					-
No	9858	19.7	1.00		
Yes	40171	80.3	1.05 (0.92-1.20)	0.491	
***Current Alcohol use (units per week)***					
None	11242	22.5	1.00		<0.001
Less than 1 unit	8741	17.5	0.90 (0.77-1.03)	0.127	
1-3 units	10288	20.6	0.71 (0.61-0.83)	<0.001	
4-6 units	7603	15.2	0.73 (0.62-0.86)	<0.001	
7-10 units	6052	12.1	0.67 (0.56-0.82)	<0.001	
11+ units	6103	12.2	0.83 (0.70-1.00)	0.049	
***Skirt size difference***					
No change	215	0.4	1.00		0.181
+1-2 sizes	3,226	6.5	0.97 (0.84-1.12)	0.690	
+3 or more sizes	8743	17.5	1.07 (0.89-1.29)	0.485	
−1-2 sizes	30,093	60.2	1.60 (1.30-1.96)	<0.001	
−3 or more sizes	7,752	15.5	1.20 (0.62-2.34)	0.592	
***Ever smoked***					-
No	27849	55.7	1.00		
Yes	22180	44.3	1.57 (1.42-1.74)	<0.001	
***History of high blood pressure***					-
No	33979	67.92	1.00		
Yes	16050	32.08	1.13 (1.01-1.26)	0.035	
***History of heart disease***					-
No	47209	94.36	1.00		
Yes	2820	5.64	1.52 (1.30-1.79)	<0.001	
***History of high blood cholesterol***					-
No	37898	75.75	1.00		
Yes	12131	24.25	0.80 (0.71-0.90)	<0.001	
***History of diabetes***					-
No	47484	94.91	1.00		
Yes	2545	5.09	1.48 (1.24-1.77)	<0.001	
***History of rheumatoid arthritis***					-
No	47626	95.2	1.00		
Yes	2403	4.8	1.12 (0.93-1.36)	0.231	
***History of osteoarthritis***					-
No	41678	83.31	1.00		
Yes	8351	16.69	0.98 (0.86-1.11)	0.720	
***History of stroke***					-
No	49268	98.48	1.00		
Yes	761	1.52	1.71 (1.33-2.22)	<0.001	
***History of osteoporosis***					-
No	46359	92.66	1.00		
Yes	3670	7.34	1.28 (1.09-1.49)	0.002	

### All-cause mortality

All associations between all-cause mortality and covariates were statistically significant with p-values <0.05, with the exception of country of birth.

All-cause mortality rate decreased with education (7.9 per 1000 person years for those with no formal education beyond 16 years versus 4.7 per 1000 person years for those with a college or university degree) (Additional file 1: Table S1). The crude association between education and mortality was statistically significant (p=<0.001 for those with no formal education) but when adjusted for all covariates this relationship was no longer significant (p=0.1) for those with no formal education) (Additional file 1: Table S2). A clear dose response relationship remained with hazard ratios from 1.04 and 1.13 for other formal education and no formal education respectively. A test for trend was statistically significant providing evidence that the less educated the individual, the higher the risk of mortality (p=<0.001).

All-cause mortality rate increased with IMD rank (5.1 per 1000 person years for the least deprived group versus 8.4 per 1000 person years for the most deprived group) (Additional file 1: Table S1). The crude association between IMD rank and mortality was significant (p=<0.001 for the most deprived group) and when adjusted for all covariates this relationship remained significant (p=0.001 for the most deprived group) with a hazard ratio of 1.45 for the most deprived group (Additional file 1: Table S3). A test for trend was significant (p=<0.001) providing evidence that the risk of mortality increases with deprivation.

Table 1 shows the association between education, IMD rank and mortality when education and IMD rank were entered simultaneously into a regression model. After adjusting for all the covariates, education was no longer significant whilst IMD rank remained significant (HR 1.42 for the most deprived group; p = 0.004) with a clear gradient.

**Figure 2:**
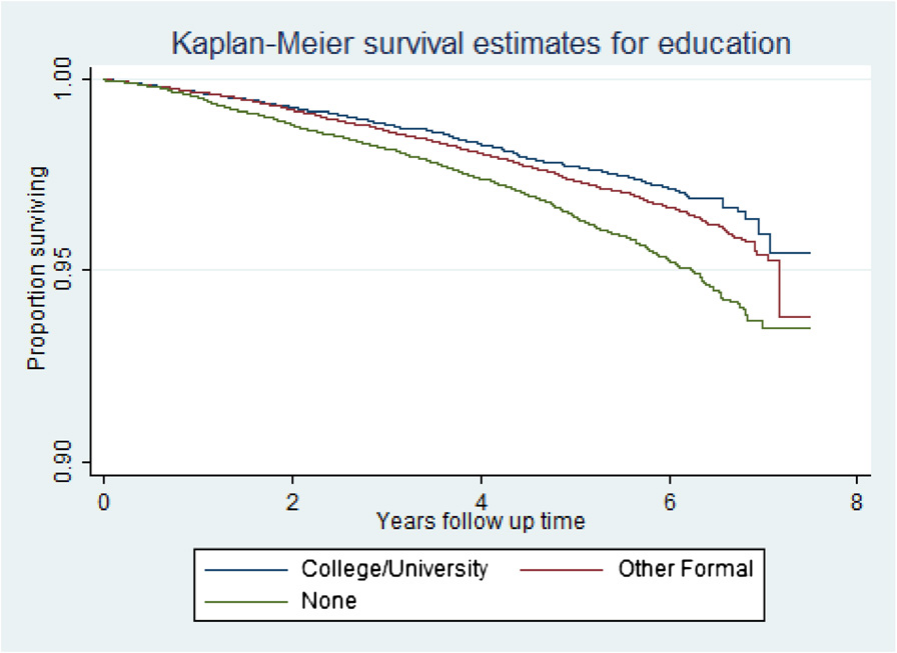
**Kaplan-Meier survival estimates for education.**

**Figure 3:**
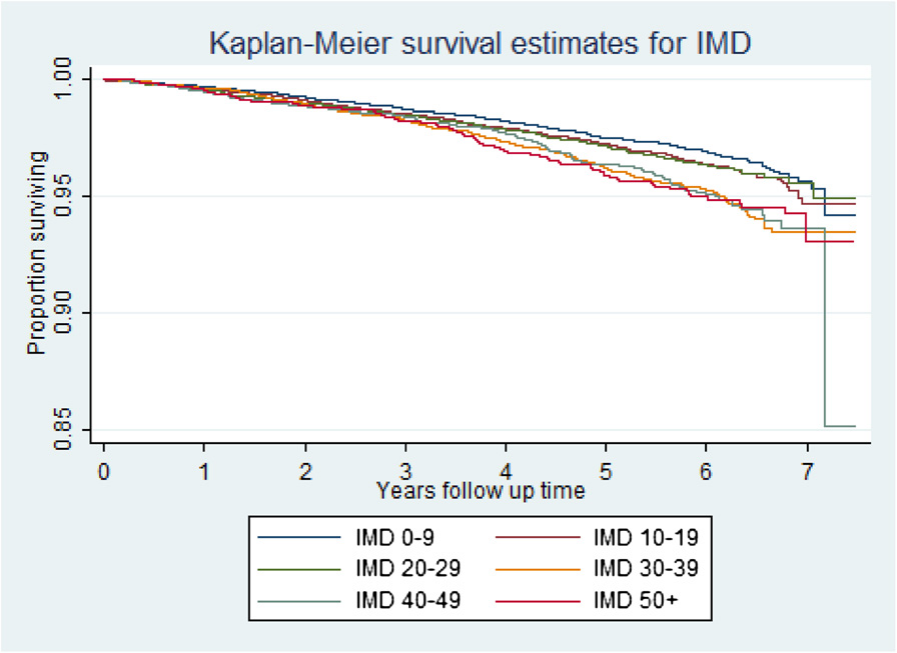
**Kaplan-Meier survival estimates for IMD.**

The Kaplan-Meier survival curves (Figures 2 and 3) show no difference in survival for either IMD or education. There is a clear gradient in survival by education and clear differentiation between the three less deprived and the three more deprived IMD groups. Proportionality was tested using the Schoenfeld residuals test and the proportional hazards assumption was not violated (p=0.256).

### Cancer mortality

Crude analysis between volunteers with no formal education compared to those with education and after adjusting for age, no formal education was significant (p=0.041 for those with no formal education), as was the test for trend (p=0.024). Age adjusted analysis for no formal education compared to college/university education had a hazard ratio of 1.22. The association between IMD rank and cancer mortality was also significant (p=0.012 for the most deprived group). For the most deprived group the age adjusted hazard ratio was 1.59 (p=0.002) and there was a significant dose response relationship between IMD rank and cancer mortality (p=0.001) (Additional file 1: Table S4).

Table 2 shows the mutually adjusted analysis model for the association between education, IMD rank and cancer mortality. Education was no longer significant (p=0.187 for those with no formal education) but there was a significant gradient (p=<0.001) with a hazard ratio of 1.14 for those with no formal education compared to those with college/university education. While the IMD rank only remained significant for groups 4, 5 and 6, the test for trend was significant (p=0.003). For the most deprived group, the age adjusted hazard ratio was 1.58 (p=0.005).

**Table 2:** **The association between cancer mortality, education and IMD rank in a mutually adjusted model, n=50,029**

***Education Level***	***Model adjusted for age HR (95% CI)***	***P-value***	***P-value for trend***
College/University	1.00		<0.001
Other formal qualification	1.06 (0.89-1.27)	0.512	
None	1.14 (0.94-1.38)	0.187	
***IMD rank***	***Model adjusted for age HR (95% CI)***	***P-value***	***P-value for trend***
1=Least deprived (0–9)	1.00		0.003
2 (10-19)	1.16 (0.98-1.36)	0.082	
3 (20-29)	1.10 (0.90-1.36)	0.352	
4 (30-39)	1.35 (1.06-1.71)	0.013	
5 (40-49)	1.65 (1.25-2.16)	<0.001	
6=Most deprived (50+)	1.58 (1.14-2.08)	0.005	

### CVD mortality

Crude analysis comparing volunteers with no formal education compared to those with education and after adjusting for age, no formal education was statistically significant (p=0.034 for those with no formal education), as was the test for trend (p=0.008). Age adjusted analysis for no formal education had a hazard ratio of 1.44. The association between IMD rank and CVD mortality showed a clear dose response relationship. The test for trend was significant (p=<0.001) and hazard ratio for the most deprived group compared to the least deprived group was 1.63 after adjusting for age (Additional file 1: Table S5).

Table 3 shows the mutually adjusted analysis model for the association between education, IMD rank and CVD mortality. Education is no longer statistically significant (p=0.134 for those with no formal education) but the test for trend was significant (p=<0.001) showing a social gradient and a hazard ratio of 1.30 for those with no formal education compared to those with college/university education. IMD rank showed a significant trend (p=0.018) towards increasing mortality but only groups 3 and 4 remained significant (p=0.001 and p=<0.001 respectively). For the most deprived group the age adjusted hazard ratio was 1.48 (p=0.199).

**Table 3:** **The association between cardiovascular disease mortality, education and IMD rank in a mutually adjusted model, n=50,029**

***Education Level***	***Model adjusted for age HR (95% CI)***	***P-value***	***P-value for trend***
College/University	1.00		<0.001
Other formal qualification	1.02 (0.73-1.42)	0.923	
None	1.30 (0.92-1.82)	0.134	
***IMD rank***	***Model adjusted for age HR (95% CI)***	***P-value***	***P-value for trend***
1=Least deprived (0–9)	1.00		0.018
2 (10-19)	1.32 (0.97-1.79)	0.075	
3 (20-29)	1.76 (1.25-2.47)	0.001	
4 (30-39)	2.32 (1.60-3.39)	<0.001	
5 (40-49)	1.40 (0.81-2.42)	0.224	
6=Most deprived (50+)	1.48 (0.81-2.69)	0.199	

### Sensitivity analysis

Missing data for all variables was 8.3%. Sensitivity analyses looking at the entire cohort (79,006) and those who had completed the follow-up questionnaire (53,886) showed similar proportions to the study sample (50,029). Mortality rates were similar as were the results of Cox regression although the associations were stronger and the confidence intervals smaller in the analysis involving the entire cohort (79,006). This was most likely due to the larger sample size and longer follow-up time resulting in a larger number of person-years available for analysis.

## Discussion

### Summary of findings

This is one of the largest studies examining the association between individual-level education and area-level indicators of socioeconomic position and all-cause and cause-specific mortality that we are aware of. Our analysis of over 50,000 older women residing in the UK between 2001–12 found both IMD rank and education to be positively associated with all-cause mortality with a clear dose response relationship. IMD rank remained positively associated with all-cause mortality after taking into account a wide range of potential confounders such as age, smoking, alcohol consumption, BMI, co-morbidities and mutually adjusting for education. There was a clear gradient even within the context of a screening trial with a significant ‘healthy volunteer effect’ [26] with the hazard ratio for all-cause mortality for those living in the most deprived areas (1.42) being higher than reported in previous studies. The survival curves (Figures 2 and 3) suggest that neither educational nor IMD differences in survival converge at later years of the study (when populations get older). We also found a statistically significant inverse trend with CVD mortality and all cancer mortality after adjusting for individual-level education. The results support increasing evidence that place of residence of individuals affects their health, [16,17,32,33] and that the IMD scale can be an effective tool to assess this effect.

### Strengths and limitations

A key strength of our study is that unlike previous studies which only examined education (individual-level variable) or IMD rank (area-level variable) as indicators of deprivation, we used both and therefore were able to account for individual social circumstances and area-level deprivation. We were able to adjust for a large number of individual-level factors such as BMI, comorbidity, smoking and alcohol use unlike analyses using national statistics. The scale of this analysis of 50,029 women was significantly larger than comparable previous studies with sample sizes ranging from 1,811–31,264 [13,25]. The fact that IMD was obtained from an external source while education was collected through postal questionnaire from trial participants and the large number of clusters (6954) ensured that the choice of method accounting for clustering of data did not substantially influence the finding, allowing a separation between the effects of IMD and education. An additional methodological strength was low proportion of missing data (8.3%). Sensitivity analysis was undertaken with imputation of the missing results as well as inclusion of the entire cohort (79,006) including those who had only completed the baseline questionnaire (24,467) with no change in results.

Certain design characteristics of our screening trial need to be considered when interpreting the current findings. While almost 1 million women were randomly invited from ‘age/sex’ registers of 27 health authorities [27], those that participated may not be entirely representative of the women living in England. Health authorities in the North East and South West England were not included as there were no trial centres in these regions. Our cohort seems to be somewhat different from the general population with 97.8% white participants compared to 85.5% of the UK population [34]. Alcohol consumption and smoking were also much higher in our cohort. Alcohol consumption (>7 units per week) was 40.4% compared to 19% (in women aged 45–64) or 6% (aged over 65) and smoking was 44.3% versus 37% in the UK population (all ages) [35,36]. The IMD scores of the invitees were higher than the national average, possibly due to an over-representation of urban centres [26]. This indicates that the 50,029 (17.3%) of those eligible and willing to participate, and included in this nested cohort study, were less deprived which could potentially bias the study. A significant ‘healthy volunteer effect’ has been reported on this cohort [26] which could have led to an underestimation of the impact of area-level deprivation on mortality. In addition, underestimation might have also resulted from inclusion of the variables in the models as confounders when they were at least in part mediators. For example, deprivation may well impact on mortality, in part, through greater access to fast food, alcohol and tobacco outlets, encouraging poor health choices, which in turn cause comorbidity and ultimately lead to higher death rates. Education was the only available indicator of personal deprivation in UKCTOCS. There is controversy whether education alone without income data is a sound indicator of individual deprivation [14,37,38].

The use of the Index of Multiple Deprivation as a predictor of health has been debated. However, the English IMD 2007 used in this analysis has been developed over the years to a high standard. It has seven complex and multifaceted domains - income and employment each contributing to 22.5% of the IMD score; barriers to housing/services, crime and living environment 9.3% each; and education and health deprivation/disability 13.5% each. While the ‘health deprivation/disability’ domain is based on years of potential life lost, comparative illness and disability ratio, measures of acute morbidity and the proportion of adults under 60 suffering from mood/anxiety disorders [39] and could contribute to the association between IMD rank and mortality, as is clear from above, it only contributes to a tenth of the score. It would have been interesting and useful to look at each domain separately but linkage to this data was not possible. Full multilevel analysis is probably the most appropriate method to answer the question of whether there are associations between area-level deprivation and mortality over and above individual-level measures. However, multilevel Cox regression using multiply imputed data was not possible in the current version of the statistical software available to the authors. We accounted for potential geographical clustering of the data by calculating standard errors using a cluster-robust estimator. We believe that this method, together with the large number of clusters, ensured that clustering did not substantially influence the results and we were able to separate between the effects of IMD and education.

### Area-level deprivation and mortality

Our study’s results show a strong association between area-level deprivation expressed by IMD and all-cause mortality which is in line with previous findings [40,41]. Multiple domains of IMD might represent several possible explanations and mechanisms that can link social disadvantage and mortality including differences in wealth and income, risky health behaviours, access to healthcare and treatment, accumulation of social disadvantage in different stages of lifecourse or psychosocial factors such as control, autonomy or trust [42]. IMD rank showed a dose response relationship on subgroup analysis of cancer and cardiovascular disease mortality, with the magnitude of effect increasing as deprivation score increased. There is much evidence to support the concept that socioeconomic status [43-45] and area-level measures of deprivation [46,47] are related to cancer survival. The association with cardiovascular mortality was not significant although there was a clear gradient in keeping with other reports [48-50]. The non-significant results may be due to the relatively low number of cause-specific deaths. In our cohort (median age 60) there were approximately three cancer deaths to every CVD death, in keeping with recent national statistics which report cancer to be the leading cause of death in those aged below 85 [51,52].

### Individual-level education and mortality

While education showed a clear social gradient with all-cause mortality in keeping with previous literature, [13,14,37,38,53,54] when mutually adjusted for IMD rank, a reduced, non-significant effect was seen (although p for trend remained significant probably due to the large sample size). This is in contrast to most other studies where education was found to be a significant predictor of mortality. However, our findings in older women are in keeping with those of Steenland et al. [19] who found that in multivariable analysis of a large US sample, education remained an important predictor only among men but not women. Others have also reported a weak association of education with mortality among women [55-57]. Variations in education level could result in variations in mortality through both access to higher paying occupations and through health literacy.

## Conclusions

Our data from UKCTOCS emphasises the importance of considering area-level indicators of socioeconomic status and supports the hypothesis that those living in more deprived areas in England have higher all-cause and cause-specific mortality rates than those living in less deprived areas. There may be several reasons for this - area-level indicators may capture community characteristics that play a role in mortality or residual confounders not adequately characterized by individual-level variables. Our analysis highlights the continued need to improve community services and living environments in deprived areas in order to reduce health inequality. As a recent Lancet editorial [58] points out, prioritising social protection in the midst of austerity is key to preventing a financial recession becoming a health recession.

## Additional file

Additional file 1
**Table S1.** Mortality rates for study sample, n = 50,029.** Table S2.** The association between all-cause mortality and education, n=50,029. **Table S3.** The association between all-cause mortality and IMD rank, n=50,029; **Table S4.** The association between cancer mortality, education and IMD rank, n=50,029. **Table S5.** The association between cardiovascular disease mortality, education and IMD rank, n=50,029.

## Abbreviations

BMI: Body Mass Index
CVD: Cardiovascular Disease
GCRC: Gynaecological Cancer Research Centre
HRT: Hormone Replacement Therapy
HSCIC: Health and Social Care Information Centre
ICD10: International Classification of Diseases 10th revision
IMD: Index of Multiple Deprivation
UKCTOCS: United Kingdom Collaborative Trial of Ovarian Cancer Screening
UCL: University College London
