# Supplementary material for: Socioeconomic indicators of health inequalities and female mortality: a nested cohort study within the United Kingdom Collaborative Trial of Ovarian Cancer Screening (UKCTOCS)
Source: BMC Public Health. 2015 Mar 17;15:253. doi: 10.1186/s12889-015-1609-5 (PMC4367890; doi:10.1186/s12889-015-1609-5)
Supplement: Additional file 1 — Table S1. Mortality rates for study sample, n = 50,029. Table S2. The association between all-cause mortality and education, n=50,029. Table S3. The association between all-cause mortality and IMD rank, n=50,029; Table S4. The association between cancer mortality, education and IMD rank, n=50,029. Table S5. The association between cardiovascular disease mortality, education and IMD rank, n=50,029. [file 12889_2015_1609_MOESM1_ESM.docx]

**Supplementary Information**

| **Table S1. Mortality rates for study sample, n = 50,029** | | | | |
| --- | --- | --- | --- | --- |
|  | **Deaths** | **Person/Years** | **Rate per 1000 person/years (C.I)** | **P-value** |
| **Ethnicity** |  |  |  | 0.015* |
| White | 1,585 | 257,445 | 6.2 (5.9-6.5) |  |
| Black | 12 | 2,309 | 5.2 (3.0-9.2) |  |
| South Asian | 1 | 1,309 | 0.8 (0.1-5.4) |  |
| Other | 6 | 2,145 | 2.8 (1.3-6.2) |  |
| **Country of Birth** |  |  |  | 0.744* |
| England | 1,447 | 236,085 | 6.1 (5.8-6.5) |  |
| Scotland | 37 | 5,310 | 7.0 (5.0-9.6) |  |
| Wales | 17 | 3,643 | 4.7 (3.0-7.5) |  |
| Northern Ireland | 8 | 1,288 | 6.2 (3.1-12.4) |  |
| Irish Republic | 17 | 3,323 | 5.1 (3.2-8.2) |  |
| Elsewhere | 78 | 13,559 | 5.8 (4.6-7.2) |  |
| **IMD Rank (1=least deprived/9=most deprived)** |  |  |  | <0.001* |
| 1 (0-9) | 449 | 87,415 | 5.1 (4.7-5.6) | <0.001** |
| 2 (10-19) | 520 | 87,790 | 5.9 (5.4-6.5) |  |
| 3 (20-29) | 247 | 41,120 | 6.0 (5.3-6.8) |  |
| 4 (30-39) | 187 | 22,626 | 8.3 (7.2-9.5) |  |
| 5 (40-49) | 104 | 12,687 | 8.2 (6.8-9.9) |  |
| 6 (50+) | 197 | 11,570 | 8.4 (6.9-9.9) |  |
| **BMI Kg/M2** |  |  |  | <0.001* |
| Underweight (11-19) | 76 | 9,677 | 7.9 (6.3-9.8) | 0.022** |
| Normal Weight (20-24) | 603 | 107,397 | 5.6 (5.2-6.1) |  |
| Overweight (25-29) | 574 | 96,898 | 5.9 (5.5-6.4) |  |
| Obese (30-39) | 314 | 45,377 | 6.9 (6.2-7.7) |  |
| Morbidly Obese (40+) | 37 | 3,858 | 9.6 (7.0-13.2) |  |
| **HRT (Current at baseline)** |  |  |  | <0.001* |
| Yes | 270 | 56,108 | 4.8 (4.3-5.4) |  |
| No | 1,334 | 207,099 | 6.4 (6.1-6.8) |  |
| **Education** |  |  |  | <0.001* |
| College/university degree or equivalent | 254 | 53,678 | 4.7 (4.2-5.4) | <0.001** |
| Other formal qualifications | 721 | 129,620 | 5.6 (5.2-6.0) |  |
| None | 629 | 79,910 | 7.9 (7.3-8.5) |  |
|  |  |  |  |  |
| * P-value for heterogeneity |  |  |  |  |
| ** P-value for trend |  |  |  |  |

| **Table S1 continued. Mortality rates for study sample, n = 50,029** | | | | | | |
| --- | --- | --- | --- | --- | --- | --- |
|  | | | **Deaths** | **Person/Years** | **Rate per 1000 person/years (C.I)** | **P-value** |
| **Alcohol (units per week)** | | |  |  |  | <0.001* |
| None | | | 511 | 58,842 | 8.7 (8.0-9.5) | <0.001** |
| Less than 1 | | | 302 | 45,665 | 6.6 (5.9-7.4) |  |
| 1-3 | | | 274 | 53,957 | 5.1 (4.5-5.7) |  |
| 4-6 | | | 200 | 40,258 | 5.0 (4.3-5.7) |  |
| 7-10 | | | 145 | 32,016 | 4.5 (3.8-5.3) |  |
| 11+ | | | 172 | 32,470 | 5.3 (4.6-6.2) |  |
| **Change in skirt size from early 20's to time of follow up** | | |  |  |  | <0.001* |
| -3 sizes or greater | | | 9 | 1,129 | 8.0 (4.2-15.3) | 0.075** |
| -1 or -2 sizes | | | 145 | 16,747 | 8.7 (7.4-10.2) |  |
| No change | | | 240 | 46,038 | 5.2 (4.6-5.9) |  |
| +1 or +2 sizes | | | 897 | 158,480 | 5.7 (5.3-6.0) |  |
| +3 sizes or greater | | | 313 | 40,814 | 7.7 (6.9-8.6) |  |
| **Smoking** | | |  |  |  | <0.001* |
| Yes | | | 874 | 117,173 | 7.5 (7.0-8.0) |  |
| No | | | 730 | 146,034 | 5.0 (4.6-5.4) |  |
| Number of years smoking | | |  |  |  | <0.001*** |
| Average number of cigarettes smoked daily | | |  |  |  | <0.001*** |
| **Self-Reported Health Conditions at Follow Up** | | | |  | |  |
| High blood pressure | | Yes | 664 | 83,277 | 8.0 (7.4-8.6) | <0.001* |
|  | | No | 940 | 179,930 | 5.2 (4.9-5.6) |  |
| Heart disease | | Yes | 200 | 14,494 | 13.8 (12.0-15.9) | <0.001* |
|  | | No | 1,404 | 248,713 | 5.6 (5.4-5.9) |  |
| High blood cholesterol | | Yes | 444 | 62,793 | 7.1 (6.4-7.8) | <0.001* |
|  | | No | 1,160 | 200,414 | 5.8 (5.5-6.1) |  |
| Diabetes | | Yes | 153 | 12,935 | 11.8 (10.1-13.9) | <0.001* |
|  | | No | 1,451 | 250,272 | 5.8 (5.5-6.1) |  |
| Rheumatoid arthritis | | Yes | 167 | 12,670 | 9.2 (7.6-11.0) | <0.001* |
|  | | No | 1,488 | 250,537 | 5.9 (5.6-6.2) |  |
| Osteoarthritis | | Yes | 113 | 43,579 | 7.1 (6.4-8.0) | 0.002* |
|  | | No | 1,293 | 219,628 | 5.9 (5.6-6.2) |  |
| Stroke | | Yes | 65 | 3,853 | 16.9 (13.2-21.5) | <0.001* |
|  | | No | 1,539 | 259,354 | 5.9 (5.6-6.2) |  |
| Osteoporosis | | Yes | 185 | 18,768 | 9.9 (8.5-11.4) | <0.001***** |
|  | | No | 1,419 | 244,439 | 5.8 (5.5-6.1) |  |
| Any of the above conditions | | Yes | 1,050 | 148,399 | 7.1 (6.7-7.5) | <0.001* |
|  | | No | 554 | 114,809 | 4.8 (4.4-5.2) |  |
| * P-value for heterogeneity | |  |  |  |  |  |
| ** P-value for trend  *** P-value (t-test) |  |  |  |  |  |  |

| **Table S2. The association between all-cause mortality and education, n=50,029** | | | |
| --- | --- | --- | --- |
| **Education level** | **Crude Hazard Ratio (95% CI)** | **P-value** | **P-value for trend** |
| College/University | 1.00 |  | <0.001 |
| Other formal qualification | 1.17 (1.02-1.35) | 0.029 |  |
| None | 1.65 (1.42-1.91) | <0.001 |  |
|  | **Model adjusted for all confounders^a^ (95% CI)** | **P-value** | **P-value for trend** |
| College/University | 1.00 |  | <0.001 |
| Other formal qualification | 1.04 (0.90-1.20) | 0.586 |  |
| None | 1.13 (0.98-1.32) | 0.100 |  |
|  |  |  |  |
| **^a^** Adjusted for age, ethnicity, BMI, HRT use, alcohol consumption, skirt size difference, smoking, high blood pressure, heart disease, high blood cholesterol, diabetes, rheumatoid arthritis, osteoarthritis, stroke and osteoporosis. | | | |

| **Table S3. The association between all-cause mortality and IMD rank, n=50,029** | | | |
| --- | --- | --- | --- |
| **IMD Rank** | **Crude Hazard Ratio (95% CI)** | **P-value** | **P-value for trend** |
| 1 = Least deprived (0-9) | 1.00 |  | <0.001 |
| 2 (10-19) | 1.16 (1.02-1.32) | 0.024 |  |
| 3 (20-29) | 1.17 (0.99-1.36) | 0.053 |  |
| 4 (30-39) | 1.58 (1.33-1.88) | <0.001 |  |
| 5 (40-49) | 1.56 (1.25-1.94) | <0.001 |  |
| 6 = Most deprived (50+) | 1.58 (1.25-2.01) | <0.001 |  |
| **IMD Rank** | **Model adjusted for all confounders ^a^ (95% CI)** | **P-value** | **P-value for trend** |
| 1 = Least deprived (0-9) | 1.00 |  | <0.001 |
| 2 (10-19) | 1.14 (1.00-1.30) | 0.040 |  |
| 3 (20-29) | 1.11 (0.95-1.30) | 0.197 |  |
| 4 (30-39) | 1.46 (1.23-1.73) | <0.001 |  |
| 5 (40-49) | 1.44 (1.16-1.79) | 0.001 |  |
| 6 = Most deprived (50+) | 1.45 (1.14-1.83) | 0.001 |  |
|  |  |  |  |
| **^a^** Adjusted for age, ethnicity, BMI, HRT use, alcohol consumption, skirt size difference, smoking, high blood pressure, heart disease, high blood cholesterol, diabetes, rheumatoid arthritis, osteoarthritis, stroke and osteoporosis. | | | |

| **Table S4. The association between cancer mortality, education and IMD rank, n=50,029** | | | |
| --- | --- | --- | --- |
| **Education level** | **Crude Hazard Ratio (95% CI)** | **P-value** | **P-value for trend** |
| College/University | 1.00 |  | <0.001 |
| Other formal qualification | 1.13 (0.95-1.36) | 0.170 |  |
| None | 1.47 (1.22-1.77) | <0.001 |  |
|  | **Model adjusted for age (95% CI)** | **P-value** | **P-value for trend** |
| College/University | 1.00 |  | 0.024 |
| Other formal qualification | 1.07 (0.89-1.28) | 0.483 |  |
| None | 1.22 (1.01-1.47) | 0.041 |  |
| **IMD rank** | **Crude Hazard Ratio (95% CI)** | **P-value** | **P-value for trend** |
| 1 = Least deprived (0-9) | 1.00 |  | <0.001 |
| 2 (10-19) | 1.31 (0.96-1.33) | 0.134 |  |
| 3 (20-29) | 1.08 (0.88-1.31) | 0.477 |  |
| 4 (30-39) | 1.32 (1.05-1.66) | 0.019 |  |
| 5 (40-49) | 1.61 (1.22-2.11) | 0.001 |  |
| 6 = Most deprived (50+) | 1.50 (1.09-1.96) | 0.012 |  |
|  | **Model adjusted for age (95% CI)** | **P-value** | **P-value for trend** |
| 1 = Least deprived (0-9) | 1.00 |  | <0.001 |
| 2 (10-19) | 1.16 (0.99-1.37) | 0.068 |  |
| 3 (20-29) | 1.12 (0.91-1.37) | 0.278 |  |
| 4 (30-39) | 1.38 (1.09-1.74) | 0.007 |  |
| 5 (40-49) | 1.69 (1.29-2.22) | <0.001 |  |
| 6 = Most deprived (50+) | 1.59 (1.19-2.14) | 0.002 |  |

| **Table S5. The association between cardiovascular disease mortality, education and IMD rank, n=50,029** | | | |
| --- | --- | --- | --- |
| **Education Level** | **Crude Hazard Ratio (95% CI)** | **P-value** | **P-value for trend** |
| College/University | 1.00 |  | <0.001 |
| Other formal qualification | 1.12 (0.80-1.57) | 0.506 |  |
| None | 1.92 (1.37-2.70) | <0.001 |  |
|  | **Model adjusted for age (95% CI)** | **P-value** | **P-value for trend** |
| College/University | 1.00 |  | 0.008 |
| Other formal qualification | 1.02 (0.73-1.43) | 0.894 |  |
| None | 1.44 (1.03-2.03) | 0.034 |  |
| **IMD rank** | **Crude Hazard Ratio (95% CI)** | **P-value** | **P-value for trend** |
| 1 = Least deprived (0-9) | 1.00 |  | 0.001 |
| 2 (10-19) | 1.29 (0.95-1.75) | 0.104 |  |
| 3 (20-29) | 1.73 (1.23-2.44) | 0.002 |  |
| 4 (30-39) | 2.32 (1.59-3.39) | <0.001 |  |
| 5 (40-49) | 1.40 (0.81-2.42) | 0.229 |  |
| 6 = Most deprived (50+) | 1.42 (0.78-2.59) | 0.247 |  |
|  | **Model adjusted for age (95% CI)** | **P-value** | **P-value for trend** |
| 1 = Least deprived (0-9) | 1.00 |  | <0.001 |
| 2 (10-19) | 1.35 (0.99-1.83) | 0.057 |  |
| 3 (20-29) | 1.84 (1.31-2.60) | <0.001 |  |
| 4 (30-39) | 2.48 (1.70-3.62) | <0.001 |  |
| 5 (40-49) | 1.52 (0.88-2.62) | 0.136 |  |
| 6 = Most deprived (50+) | 1.63 (0.90-2.96) | 0.106 |  |
